# Prevalence and risk factors of hepatitis B and C viruses among haemodialysis patients in Gaza strip, Palestine

**DOI:** 10.1186/1743-422X-7-210

**Published:** 2010-09-01

**Authors:** Abed El-kader Y El-Ottol, Abdelraouf A Elmanama, Basim M Ayesh

**Affiliations:** 1 Microbiology Department, Central Laboratory, Al-Shifa Hospital, Gaza, Palestine; 2 Medical Technology Department, Islamic University, Gaza, Palestine; 3 Biology Department, Al-Aqsa University, Gaza, Palestine

## Abstract

**Background:**

The prevalence of hepatitis B virus (HBV) and hepatitis C virus (HCV) and its associated risk factors among haemodialysis (HD) patients in Gaza strip was investigated using serological and molecular techniques.

**Results:**

The overall prevalence of HBV among the four HD centers was 8.1%. The main risk factors were HD center (p = 0.05), history of blood transfusion (p < 0.01), and treatment abroad (p = 0.01). The overall prevalence of HCV among the four HD centers was 22%. The main risk factors were HD center (p < 0.01), time duration on HD (p < 0.01), history of blood transfusion (p < 0.01), treatment abroad (p < 0.01), and history of blood transfusion abroad (p < 0.01). Serum aminotransferases levels decreased in HD patients compared with normal population but still there was a direct association between the activity of liver enzymes and both HBV (p < 0.01) and HCV (p < 0.01) infection.

**Conclusion:**

The much higher prevalence of Hepatitis viruses among HD patients compared to the normal population of Gaza strip indicates a causative relation between HD and hepatitis viruses transmission. Therefore extremely careful observation of preventive infection control measures is essential to limit Hepatitis viruses' transmission in HD centers.

## Introduction

Hepatitis B virus (HBV) and hepatitis C virus (HCV) infections are important causes of morbidity and mortality among haemodialysis (HD) patients and pose problems in the management of patients in the renal dialysis units, because chronic renal failure patients do not clear these viral infections efficiently [1]. In Arab countries, the prevalence of chronic HBsAg positivity among HD patients ranged from 2% in Morocco, to 11.8% in Bahrain [2-5]. Also In Arab countries the prevalence of HCV antibodies among HD patients has been reported to range from 27% in Lebanon to 75% in Syria [6-9].

However there are strong indications that studies of HD patients which rely solely on serological screening could underestimate the prevalence of HCV infection. Partial immunesuppression in these patients, resulting in poor antibody response may be a contributing factor [10]. Such shortcomings could be overcome by determining HCV RNA, which may be required to identify all infected patients [11].

No documented data or previous studies have been reported on the prevalence of hepatitis viruses among HD patients in Palestine and to the best of our knowledge this study is the first to address this issue. Therefore the main objective of this study was to estimate the prevalence of HBV and HCV among HD patients in Gaza strip and address the major risk factors for transmission of these viruses among HD patients.

## Materials and methods

### Patients

All of the four governmental HD centers of Gaza strip were included in this study, and a total of 246 patients were tested during August to September 2007. The study principles and protocols were submitted and approved by the committee of Helsinki, verbal consent was obtained from each patient after the principle of the study and its possible outcomes were explained to all subjects.

All personal information of the study subjects and result were dealt with in confidentiality. A close ended and multiple choice based questionnaire was completed by the researcher via patient interview to ensure proper data collection and prevent any misunderstanding.

### Samples collection

Two blood samples were collected from each patient, in plain tube, prior to dialysis to prevent the interference of heparin with downstream applications. Serum from the first tube was tested within two hours for ALT, AST, HBsAg, and anti-HCV antibodies. Serum from the second tube was frozen at -70°C in a sterile, DNAse, RNAse free tightly capped tube until used for PCR analysis.

### Virology

For HBsAg determination, Axsym HBsAg version 2.0 kit (Abbott, USA) was used, Non reactive samples were considered negative for HBsAg and not tested further, while a reactive sample was retested to confirm the result; a repeatedly reactive sample was considered positive and not further tested.

For anti-HCV antibodies determination, Axsym HCV version 3.0 kit (Abbott, USA) was used. Non reactive samples were considered negative for HCV, while reactive samples were retested to confirm the result and repeatedly reactive samples were considered positive. Serologically positive HCV samples were tested individually by nested RT-PCR technique, while negative samples were pooled in batches of ten and tested by the same technique.

### Chemistry

Serum alanine aminotransferase (ALT) and Serum aspartate aminotransferase (AST) levels were analyzed for all samples by using Diasys, (Germany) reagents, the upper limit of normal for ALT and AST were set at 40 IU/ml and 37 IU/ml respectively [12].

### Pooling of Serum Samples

A pooling strategy was developed and tested in this study, in which ten serum samples from different patients (negative anti-HCV) were pooled together in one tube. Two hundred μl serum from each sample were combined together in a single 2 ml microcentrifuge. The tubes were ultracentrifuged for two hours at (21,000 × g) and cooling at 4°C. A pellet was visible and the supernatant was reduced to approximately 150 μl by removal of most of the liquid. The pellet was resuspended in the remaining serum and viral RNA was extracted for PCR amplification. The samples of a positive pool were either reanalyzed individually or subdivided into 3 smaller pools.

### Molecular screening for HCV RNA

Viral RNA was extracted from serum samples using the QIAamp viral RNA extraction kit according to the manufacturer recommendation (Qiagen, Germany). Both cDNA synthesis and PCR amplification of the target sequences were performed in a single tube using the one step RT-PCR kit according to the manufacturer instructions (Qiagen, Germany). The primers were designed to specifically bind the 5'UTR of the HCV genome that is highly conserved among the different genotypes. Primer sequences were as follows: sense external primer (**5'**-CCCTGTGAGGAACT**W**CTGTCTTCACGC**-3')**; antisense external primer (**5'**-G GTGCACGGTCTACGAGACCT-**3')**; sense internal primer (**5'**-TCTAGCCATGGC GTTAG T**RY**GAGTGT-**3')**; and antisense internal primer (**5'**-CACTCGCAAGCA CCCTATCA GGCAGT-**3', W **= A or T, **R **= A or G, **Y **= T or C) [13].

The reactions were carried out in 25 μl volumes using 10 μl RNA in the presence of 0.6 μM of each HCV external primer, 400 μM of each dNTP and 5 units RNase inhibitor. The reaction cycling conditions were: 1 cycle at 50°C for 30 minutes, 1 cycle at 95°C for 15 minutes followed by 40 cycles of 95°C for one minute, 55°C for one minute and 72°C for one minute. Finally the reactions were allowed to complete at 72°C for 10 minutes and held at 4°C.

In all cases a second nested PCR was performed to improve the detection capacity and specificity of the PCR test. The second PCR reactions were carried out in 25 μl volumes using 5 μl DNA template from 1^st ^PCR in the presence of 1× PCR master mix (Promega, USA) and 0.4 μM of each HCV internal primers. The reaction cycling conditions were 1 cycle at 95°C for 5 minutes, followed by 34 cycles of 95°C for one minute, 55°C for one minute and 72°C for one minute. Finally the reactions were allowed to complete at 72°C for 10 minutes and hold at 4°C.

The products were analyzed by 2% agarose gel with 100 bp ladder and stained with ethidium bromide. The appearance of a 298 bp and 235 bp bands was considered a positive results for the fist and the second PCR respectively.

### Statistical analysis

The data was collected, summarized, tabulated and analyzed using the statistical package for social sciences (SPSS) V.13 software. Differences in proportions were assessed by a chi-square test and mean comparisons by the t-test, p-value < 0.05 was considered statistically significant.

## Results

During the study, 131 patients from Al-Shifa hospital in the center of Gaza City, 30 patients from Shuhada'a Al-Aqsa Hospital in Dair El-Balah, 52 patients from Nasser hospital in Khan Yunis city, and 34 patients from Abu-Yousef Al-Najar Hospital in Rafah city were included. From the 246 patients tested, there were 131 male and 115 females. The Patients ages ranged from 6 to 80 years, and the mean age for all patients was 46.7 years with standard deviation (SD) ± 17.5 years.

The overall prevalence of HBV among HD patients in Gaza strip was found to be 8.1%. The highest percentage was11.5% in Al-Shifa hospital, followed by 9.6% in Nasser hospital, and no cases were detected in both Shuhada'a Al-Aqsa and Abu-Yousef Al-Najar Hospitals.

Analysis of risk factors showed that there was a highly statistically significant relationship between HBV infection and age of the patient (p = 0.04). The mean age for HBV positive patients was 39.2 ± 14.6 year, while for HBV negative patients was 47.4 ± 17.6 year. Male patients were found to be more susceptible to HBV than female patients (p = 0.01) (Table 1).

**Table 1:** Relationship between HBV and HCV infection and Social factors.

Variable		HBV Positive	HBV Negative	HCV Positive	HCV Negative
Age	Mean Age	39.2 ± 14.6	47.4 ± 17.6	43.4 ± 15.5	47.6 ± 17.9
	
	p Value	0.04		0.11	

Patient Sex	Male	16 (12.2%)	115 (87.8%)	30 (22.9%)	101 (77.1%)
	
	Female	4 (34.8%)	111 (96.5%)	24 (20.9%)	91 (79.1%)
	
	p Value	0.01		0.70	

Education Level	Illiterate	4 (6.2%)	61 (93.8%)	7 (10.8%)	58 (89.2%)
	
	Elementary	4 (8.2%)	45 (91.8%)	11 (22.4%)	38 (77.6%)
	
	Prep	6 (10.2%)	53 (89.8%)	22 (37.3%)	37 (62.7%)
	
	Secondary	4 (7.8%)	47 (92.2%)	7 (13.7%)	44 (86.3%)
	
	University	2 (9.1%)	20 (90.9%)	7 (31.8%)	15 (68.2%)
	
	p value	0.9		< 0.01	

Smoking	Yes	2 (12.5%)	14 (87.5%)	8 (50%)	8 (50%)
	
	No	18 (7.8%)	212 (92.1%)	46 (20%)	184 (80%)
	
	p Value	0. 5		0.01	

Other risk factors with statistically significant relation were, the HD center at which patients were treated (p = 0.05); the number of transfused blood units (p < 0.01); and history of treatment Abroad (p = 0.01) (Table 2). HBV was found to have a strong relationship with ALT and AST levels. HBV positive patients had mean ALT value of 22.4 ± 20.9 IU/ml while negative patients 13.4 ± 11.8 IU/ml (p < 0.01); Also HBV positive patients had AST mean value of 23.5 ±15.3 IU/ml while negative HBV patients 15.2 ± 11.2 IU/ml (p < 0.01) (Table 3).

**Table 2:** Relationship between HBV, HCV infection, and factors related to HD.

Variable		HBV Positive (%)	HBV Negative (%)	HCV Positive (%)	HCV Negative (%)
HD Center	Al-Shifa	15 (11.5)	115 (88.5)	28 (21.5)	102 (78.5)
	
	Al-Aqsa	0 (0)	30 (100)	11(36.7)	19 (63.3)
	
	Nasser	5 (9.6)	47 (90.4)	15(28.8)	37 (71.2)
	
	Al-Najar	0 (0)	34 (100)	0 (0)	34 (100)
	
	p Value	0.05		< 0.01	

Time Duration on HD (month)	Mean Time	37.7	34.3	49.6	30.3
	
	p value	0.68		< 0.01	

Number of Blood Units Transfused	0	7 (6.8)	96 (93.2)	19 (18.4)	84 (81.6)
	
	1- 5	4 (6.7)	56 (93.3)	6 (10)	54 (90)
	
	5-10	2 (4.4)	43 (95.6)	9 (20)	36 (80)
	
	10-15	1 (5)	19 (95)	10 (50)	10 (50)
	
	< 15	6 (33.3)	12(66.7)	10 (55.6)	8 (44.4)
	
	p value	< 0.01		< 0.01	

Number of Patients Treated Abroad	Yes	18 (11.8)	134 (88.2)	45 (29.6)	107 (70.4)
	
	No	2 (2.1)	92 (97.8)	9 (9.6)	85 (90.4)
	
	p value	0.01		< 0.01	

Surgical Operation Abroad from the152 patients treated abroad	yes	14 (11.5)	108 (88.5)	36 (29.5)	86 (70.5)
	
	no	4 (13.3)	26 (86.7)	9 (30)	21 (70)
	
	p value	0.7		0.95	

Blood Transfusion Abroad from the 152 patients treated abroad	yes	9 (12.9)	61 (87.1)	29 (41.4)	41 (58.6)
	
	no	9 (11)	73 (89)	16 (19.5)	66 (80.5)
	
	p value	0.70		< 0.01	

**Table 3:** Relationship between HBV, HCV infection and liver enzymes among HD patients.

**Liver Enzymes**		**HBV Positive**	**HBV Negative**	**HCV Positive**	**HCV Negative**
	
	ALT mean	22.4	13.4	19.9	12.5
	
	p Value	< 0.01		< 0.01	
	
	AST mean	23.5	15.2	22.9	13.9
	
	p Value	< 0.01		< 0.01	

On the other hand, no statistically significant relationship was found between HBV infection and the level of education (p = 0.9); smoking (p = 0.51) (Table 1); HD duration time (p = 0.68); blood transfusion abroad (p = 0.7); and surgical operation abroad ((p = 0.7 ) (Table 2).

The prevalence of anti-HCV was 17.9%. The highest percentage was 26.7% in Al-Aqsa Hospital; followed by 23.1% in Nasser hospital, then 18.5% in Al-Shifa hospital, and 0% in Al-Najar Hospital (Table 4).The prevalence of HCV RNA was 19.1%. The highest percentage was 36.7% in Al-Aqsa Hospital, then 23.1% in Nasser hospital, 18.5% in Al-Shifa hospital, and 0% in Al-Najar Hospital (Table 4).

**Table 4:** Overall prevalence of HCV among HD patients in Gaza Strip.

Character	HCV RNA positive	HCV RNA negative	Total
anti- HCV positive	37 (15%)	7 (2.8%)	44 (17.9%)

anti- HCV negative	10 (4.1%)	192 (78%)	202 (82.1%)

Total	47 (19.1%)	199 (80.9%)	246 (100%)

By combining the results of both serological and molecular methods, 22% of patients were found to be HCV positive. 68.5% of the positive patient samples were detected by the two methods, 18.5% of the positive patient samples by nested RT-PCR method alone and 13% of the positive patient samples by serology method alone (Table 4).

The highest prevalence of HCV was found at Al-Aqsa Hospital (36.7%), followed by Nasser hospital (28.8%), then Al-Shifa hospital (21.5%), and Al-Najar Hospital (0%). Four cases (1.6%) were identified as positive for both HBV and HCV, two cases at Al-Shifa center, and the other two cases at Nasser hospital.

For HCV infection a highly statistically significant relationship was found between HCV infection and Education level (p < 0.01). Most patients with HCV were found in the preparatory level (40.7%) followed by the Elementary level (20.4 %). Smoker patients appear to be more susceptible to HCV than non smokers (p < 0.01) (Table 1).

Other risk factors for HCV include HD centers (< 0.01), history of blood transfusion (p < 0.01), history of treatment abroad (p < 0.01), blood transfusion abroad (p < 0.01) and time duration on HD (p < 0.01). The mean time duration for HCV negative patients was 30.3 ± 33.7 months while for positive HCV was 49.6 ± 42.4 month; (Table 2).

HCV also was found to have a strong relationship with ALT and AST levels, Positive HCV patients have mean ALT value of 19.9 ± 19.1 IU/ml while negative HCV patients has ALT mean value of 12.53 ± 10.3 IU/ml (p < 0.01). Also positive HCV patients had AST mean value of 22.9 ± 16.9 IU/ml while negative HCV patients has AST mean value of 13.9 ± 9.00 IU/ml (p < 0.01) (Table 3).

No statistically significant relationship was found between HCV and patient sex (p = 0.70) and age (p = 0.11) (Table 1), and surgical operation abroad (p-0.95) (Table 2).

## Discussion

Viral hepatitis remains a major hazard for both patients and medical staff of HD units [14]. In this study, the overall prevalence of HBV in HD patients in Gaza strip was 8.1%. This prevalence higher than in other neighboring countries like Jordan (5.9%) and lower than others such as Saudi Arabia (10%) and Bahrain (11.8%) [3-5].

Statistically significant differences in prevalence between the four HD centers are obvious (p = 0.05). Such a result was also observed in a previously published research in Jordan [3]. This difference in prevalence may be due to variation in the degree of implementation of the universal precautions to prevent nosocomial transmission.

In our study no statistically significant relationship was found between HBV and duration on HD, this observation was in agreement with a previous report in Moldavia [15], and in disagreement with another in Jordan [3] In our study, the prevalence of HBV was increased significantly with increasing the number of blood units transfused (p < 0.01), This is in agreement with a previous study in Brazil [16], while another study in Jordan showed no relationship [3].

In general, most patients of Gaza strip with chronic diseases or who are in need for major surgeries are treated abroad. Also some patients during their treatment undergo HD. We found a statistically significant relationship between treatment abroad and HBV infection (p = 0.01). A previous study in India showed that traveling abroad is associated with increased risk of hepatitis virus infection and this risk is expected to be increased when traveling to endemic areas [17].

No statistically significant relationship was found between HBV prevalence and surgical operation or blood transfusion abroad. This result contradicts results from a previous work in Brazil that showed that surgery is a risk factor for acquiring hepatitis infection in the general population [18]. This contradiction may be due to differences in the type of surgery, the level of medical services and other factors related to the countries were surgeries are performed.

A statistically significant relationship was found between HBV infection and age of the patients, as patients less than 40 years old were found more susceptible to HBV than older patients. This is in agreement with a study done in Gaza among the general population, which found that the highest percentage of HBsAg among age group of 30 to 39 years [19]. The results also showed that male HD patients had higher HBV prevalence than females. This may be related to the fact that males in Palestine are more socially active than female. Furthermore, they are more exposed to male-related risk factors for HBV than female (e.g. hairdressing and circumcision). This result is in agreement with study in general population of Lebnon [20].

The prevalence of anti-HCV among HD patients was 17.9%, This anti-HCV prevalence was lower than Tunisia (19.1%), Lebanon (27%), Jordan (34.6%), and Syria (75%) [21,6,7,9]. Partial immunosuppression in HD patients resulting in a poor antibody response to Hepatitis viruses' infection [10] makes serological screening of HCV underestimate. Such short coming could be overcome by detecting HCV RNA [11].

In this study HCV RNA was detected in 19.1% of HD patients, this result is lower than some countries such as Brazil (30.6%) and Greece (31.7%) [22,23]. HCV RNA was detected in only 84.1% of the anti-HCV positive patients. This deviation between PCR and ELISA results was also previously reported in many studies like in Syria (87.5%), Jordan (30.6%), and Tunisia (72.3%) [9,7,16]. The possible explanations for this Difference in percentage of viraemic patients includes false positive serology, intermittent viraemia, or the level of viraemia in is below the lower limit of PCR detection [7]. Also the viral load is relatively low in this group of patients and long term maintenance HD decreases the HCV RNA level but doesn't produce clearance of viraemia [24].

On the other hand, HCV RNA was detected in serum of 10 patients of the anti-HCV negative (4.95%). Again, several studies have shown that serological assays alone are not sufficient for diagnosis of HCV infection and the detection of HCV RNA is required to identify all infected patients [25]. So detection of HCV RNA is more reliable than serology in detecting ongoing HCV infection in HD patients who may not mount an adequate antibody response [25]. Our results among seronegative patients were higher than studies in center of Recife, Brazil (0%) [26], but lower than another study in central Brazil (10.3%) [27]. The overall prevalence of HCV among Gazian HD patients thus is (22 %). This is higher than in Germany in which the overall prevalence was (3%) [28] and lower than in Brazil (46.7%) [29].

HCV prevalence was different between HD centers, and as stated earlier, this difference in prevalence may be due to different degrees of commitment to universal precautions taken in each center, the same result was shown in previous studies in Jordan and Tunisia [7,21].

Duration on HD was found to be a statistically significant risk factor for HCV infection in HD setting. The risk of HCV infection increased with increasing the time duration. In HD population under investigation, other studies from different regions in the world have shown similar results [30].

This effect may be due to nosocomial transmission of HCV as indicated by other studies, epidemiological and molecular studies have shown the role of HD environment for dissemination of HCV between patients [31,32]. Actually in our study it was not unusual to find staff taking care of susceptible and infected patients in the same shift. Additionally they didn't routinely discard gloves after use; this practice may facilitate the dissemination of HCV RNA between HD patients.

Blood transfusion was found to be a highly statistically significant risk factor in this study. The risk increased with the increase in the number of blood units which were transfused (p < 0.01). This result was in agreement with other study in USA[33].

No relationship was found between HCV prevalence and sex of the patients, this was in agreement with other studies [34]. Also no statistically significant relationship was found between HCV prevalence and patient's age; this finding is supported by other study [34].

A direct statistically significant relationship was found between HCV and education level in which most of HCV positive patients were in the preparatory level followed by elementary. Previous studies among general population worldwide showed that HCV is prevalent among less educated level people [35].

In this study a direct relationship was found between HCV prevalence and smoking, in which smoker patients are more susceptible to HCV than non smokers. This may be due to bad practices in Gaza strip where people are smoking water pipe (Shisha) in groups. Water pipe is passed from one person to another; the mouth piece maybe wiped but is not changed between users, which theoretically result in exposure to blood from individuals with gingivitis for example; Also smokers are highly associated with increased visit to outpatient physician and hospital services, a result that was indicated in reports among general population.

The striking difference in prevalence and risk factors seen between HBV and HCV, with similar molecular size and transmission routes in general and the HD population, was also described by others [36].

Co-infection with both HBV and HCV was reported in 4 patients (1.6%) of the total HD population which is low when compared with other study in Moldavia [15].

In our study a direct and strong association between the activity of liver enzymes and both HBV and HCV prevalence was found. Previous studies suggested that ALT levels, even that not exceeding upper limit of normal are higher in anti-HCV positive patients in comparison with anti-HCV negative patients [37,38]. Therefore recriteria for normal reference of ALT and AST is proposed for HD population. On the other hand, other study suggested that serum aminotransferases aren't reliable marker for HCV screening or for evaluation of hepatitis activity in HD patients since they are frequently normal [39].

## Conclusion

Hepatitis B and C viruses' infection are very frequent among HD patients in Gaza strip as in many regions of the world. The HD center, blood transfusion and treatment abroad appear to be the major risk factors for both viruses.

For HBV infection other risk factors included, younger age and male patients. Further studies among total population are needed to investigate this factor. For HCV other risk factors for infection include, the duration on HD, smoking, and the level of education.

The much higher prevalence of Hepatitis viruses among HD patients compared to the normal population of Gaza strip indicates a causative relation between HD and hepatitis viruses transmission.

Therefore extremely careful observation of preventive infection control measures is essential to limit Hepatitis viruses' transmission in HD centers.

## Competing interests

The authors declare that they have no competing interests.

## Authors' contributions

AYO participated in the design of the study; carried out and personally financed the studies; performed the statistical analysis; and drafted the manuscript. AAM participated in the design of the study; supervision on the theoretical and practical work. BMA participated in the design of the study; and supervised on the molecular genetic studies and the statistical analysis. All authors have read and approved the final manuscript.

## References

[B1] Saha D Agarwal SK Hepatitis and HIV infection during haemodialysis J Indian Med Assoc 2001 99 4 194 199 203,213 11666025

[B2] Boulaajaj K Elomari Y Elmaliki B Madkouri B Zaid D Benchemsi N Prevalence of hepatitis C, hepatitis B and HIV infection among haemodialysis patients in Ibn-Rochd university hospital, Casablanca Nephrol Ther 2005 1 5 274 284 16895696 10.1016/j.nephro.2005.06.012

[B3] Al Hijazat M Ajlouni Y Hepatitis B infection among patients receiving chronic hemodialysis at the royal medical services in Jordan Saudi J Kidney Dis Transpl 2008 19 2 260 267 18310881

[B4] Khan LA Khan SA Prevalence of hepatitis B and C markers in patients on maintenance hemodialysis in Najran Saudi Med J 2001 22 7 641 642 11479651

[B5] Almawi WY Qadi AA Tamim H Ameen G Bu-Ali A Arrayid S Abou Jaoude MM Seroprevalence of hepatitis C virus and hepatitis B virus among dialysis patients in Bahrain and Saudi Arabia Transplant Proc 2004 36 6 1824 1826 10.1016/j.transproceed.2004.07.019 15350487

[B6] Naman RE Mansour I Klayme S Khalil G Hepatitis C virus in hemodialysis patients and blood donors in Lebanon J Med Liban 1996 44 1 4 9 8965318

[B7] Bdour S Hepatitis C virus infection in Jordanian haemodialysis units: serological diagnosis and genotyping J Med Microbiol 2002 51 8 700 704 12171303 10.1099/0022-1317-51-8-700

[B8] Hachicha J Hammami A Masmoudi H Ben Hmida M Karray H Kharrat M Kammoun F Jarraya A Viral hepatitis C in chronic hemodialysis patients in southern Tunisia, prevalence and risk factor Ann Med Interne 1995 146 5 295 298 8526312

[B9] Abdulkarim AS Zein NN Germer JJ Kolbert CP Kabbani L Krajnik KL Hola A Agha MN Tourogman M Persing DH Hepatitis C virus genotype and hepatitis G virus in hemodialysis patients from Syria: identification of two novel hepatitis C virus subtypes Am J Trop Med Hyg 1998 59 4 571 576 9790432 10.4269/ajtmh.1998.59.571

[B10] Goldbloom SE Reed WP Host defenses and immunologic alterations associated with chronic hemodialysis Ann Intern Med 1980 93 4 597 613 7001976 10.7326/0003-4819-93-4-597

[B11] Fabrizi F Martin P Dixit V Brezina M Russell J Conrad A Schmid P Gerosa S Gitnick G Detection of de novo hepatitis C virus infection by polymerase chain reaction in hemodialysis patients Am J Nephrol 1999 19 3 383 388 10.1159/000013482 10393375

[B12] Ministry Of Health Palestinian clinical laboratory tests guide MOH 2005

[B13] Stuyver L Rossau R Wyseur A Duhamel M Vanderborght B Van Heuverswyn H Maertens G Typing of hepatitis C virus isolates and characterization of new subtypes using a line probe assay J Gen Virol 1993 74 pt 6 1093 1102 10.1099/0022-1317-74-6-1093 8389799

[B14] Feher T Ambuhi PM Chronic hepatitis virus infections in patients on renal replacement therapy Nephrol Dial Transplant 2004 19 5 1049 1053 10.1093/ndt/gfh080 15004267

[B15] Covic A Iancu L Apetrei C Scripcaru D Volovat C Mititiuc I Covic M Hepatits virus infection in haemodialysis patients from Moldavia Nephrol Dial Transplant 1999 14 1 40 45 10.1093/ndt/14.1.40 10052474

[B16] Busek SU Babá EH Tavares Filho HA Pimenta L Salomão A Corrêa-Oliveira R Oliveira GC Hepatitis C and Hepatitis B Virus Infection in Different Hemodialysis Units in Belo Horizonte, Minas Gerais, Brazil Mem Inst Oswaldo Cruz 2002 97 6 775 778 10.1590/S0074-02762002000600003 12386694

[B17] Jauréguiberry S Grandière-Pérez L Ansart S Laklache H Métivier S Caumes E Acute hepatitis C virus infection after a travel in India J Travel Med 2005 12 1 55 56 15996468 10.2310/7060.2005.00010

[B18] Busek S Oliveira G Molecular epidemiology of the hepatitis C virus in Brazil Genet Mol Res 2003 2 1 117 123 12917808

[B19] Yassin K Awad R Tebi AJ Queder A Laaser U Prevalence and risk factors of HBsAg in Gaza: Implications for prevention and control J Infect 2002 44 4 252 256 10.1053/jinf.2001.0998 12099733

[B20] Kalaajieh W Deeaoui M Chbani-Rima A Epidemiology of acute hepatitis B infection in Lebnon Med Mal Infect 2002 32 7 382 386 10.1016/S0399-077X(02)00385-2

[B21] Ayed K Gorgi Y Ben Abdallah T Aouadi H Jendoubi-Ayed S Sfar I Makni H Hepatitis C virus infection in hemodialysis patients from Tunisia: national survey by serologic and molecular methods Transplant Proc 2003 35 7 2573 2575 10.1016/j.transproceed.2003.09.005 14612022

[B22] Carneiro MA Martins RM Teles SA Silva SA Lopes CL Cardoso DD Vanderborght BO Yoshida CF Hepatitis C prevalence and risk factors in hemodialysis patients in central Brazil: a survey by polymerase chain reaction and serological methods Mem Inst Oswaldo Cruz 2001 96 6 765 769 10.1590/S0074-02762001000600003 11562698

[B23] Rigopoulou EI Stefanidis I Liaskos C Zervou EK Rizos C Mina P Zachou K Syrganis C Patsidis E Kyriakopoulos G Sdrakas L Tsianas N Dalekos GN HCV RNA qualitative assay based on transcription mediated amplification improves the detection of hepatitis C virus infection in patients on hemodialysis: Results from five hemodialysis units in central Greece J Clin Virol 2005 34 1 81 85 10.1016/j.jcv.2005.05.007 16009596

[B24] Furusyo N Hayashi J Ariyama I Sawayama Y Etoh Y Shigematsu M Kashiwagi S Maintenance hemodialysis decreases serum hepatitis C virus (HCV) RNA levels in hemodialysis patients with chronic HCV infection Am J Gastroenterol 2000 95 2 490 496 10.1111/j.1572-0241.2000.01773.x 10685756

[B25] Bukh J Wantzin P Krogsgaard K Knudsen F Purcell RH Miller RH High prevalence of hepatitis C virus (HCV) RNA in dialysis patients: failure of commercially available antibody tests to identify a signiticant number of patients with HCV infection J Infect Dis 1993 168 6 1343 1348 7504031 10.1093/infdis/168.6.1343

[B26] Albuquerque AC Coêlho MR Lopes EP Lemos MF Moreira RC Prevalence and risk factors of hepatitis C virus infection in hemodialysis patients from one center in Recife, Brazi Mem Inst Oswaldo Cruz-Rio de Janeiro 2005 100 5 467 470 10.1590/s0074-02762005000500003 16184221

[B27] Carneiro MA Martins RM Teles SA Silva SA Lopes CL Cardoso DD Vanderborght BO Yoshida CF Hepatitis C prevalence and risk factors in hemodialysis patients in central Brazil: a survey by polymerase chain reaction and serological methods Mem Inst Oswaldo Cruz 2001 96 6 765 769 10.1590/S0074-02762001000600003 11562698

[B28] Hinrichsen H Leimenstoll G Stegen G Schrader H Fölsch UR Schmidt WE PHV Study Group Prevalence and risk factors of hepatitis C virus infection in haemodialysis patients: a multicentre study in 2796 patients Gut 2002 51 3 429 433 10.1136/gut.51.3.429 12171969 PMC1773370

[B29] Reddy GA Dakshinamurthy KV Neelaprasad P Gangadhar T Lakshmi V Prevelance of HBV and HCV dual infection in patients on hemodialysis Indian J Med Microbiol 2005 23 1 41 43 10.4103/0255-0857.13872 15928421

[B30] Irish DN Blake C Christophers J Craske JE Burnapp L Abbs IC MacMahon EM Muir P Banatvala JE Simmonds P Identification of hepatitis C seroconversion resulting from nosocomial transmission on a haemodiolysis unit: implications for infection control and laboratory screening J Med Virol 1999 59 2 135 140 10.1002/(SICI)1096-9071(199910)59:2<135::AID-JMV2>3.0.CO;2-Y 10459146

[B31] Allander T Gruber A Naghavi M Beyene A Söderström T Björkholm M Grillner L Persson MA Frequent patient to patient transmission of hepatitis C virus in a haematology ward Lancet 1995 345 8950 603 607 10.1016/S0140-6736(95)90518-9 7898176

[B32] Sartor C Brunet P Simon S Tamalet C Berland Y Drancourt M Transmission of hepatitis C virus between hemodialysis patients sharing the same machine Infect Control Hosp Epidemiol 2004 25 7 609 611 10.1086/502448 15301036

[B33] de Medina M Ashby M Schlüter V Hill M Leclerq B Pennell JP Jeffers LJ Reddy KR Schiff ER Hess G Perez GO Prevalence of hepatitis C and G virus infection in chronic hemidialysis patients Am J Kidney Dis 1998 31 2 224 226 10.1053/ajkd.1998.v31.pm9469491 9469491

[B34] Lopes EP Gouveia EC Albuquerque AC Sette LH Mello LA Moreira RC Coelho MR Determination of the cut off value of serum alanine aminotransferase in patients undergoing hemodialysis, to identify biochemical activity in patients with hepatitis C viraemia J clin Virol 2006 35 3 298 302 10.1016/j.jcv.2005.09.010 16290052

[B35] el-Sadawy M Ragab H el-Toukhy H el-Mor Ael-L Mangoud AM Eissa MH Afefy AF el-Shorbagy E Ibrahem IA Mahrous S Abdel-Monem A Sabee EI Ismail A Morsy TA Etewa S Nor Edin E Mostafa Y Abouel-Magd Y Hassan MI Lakouz K Abdel-Aziz K el-Hady G Saber M Hepatitis C virus infection at Sharkia Governorate, Egypt: seroprevalence and associated risk factors J Egypt Soc Parasitol 2004 34 1 Suppl 367 384 15124747

[B36] Dussol B Berthezène P Brunet P Roubicek C Berland Y Hepatits C virus among chronic dialysis patients in the south of France: a collaborative study Am J Kidney Dis 1995 25 3 399 404 10.1016/0272-6386(95)90100-0 7532916

[B37] Fabrizi F Lunghi G Finazzi S Colucci P Pagano A Ponticelli C Locatelli F Decreased serum aminotransferase activity in patients with chronic renal failure: impact on the detection of viral hepatitis Am J Kidney Dis 2001 38 5 1009 1015 10.1053/ajkd.2001.28590 11684554

[B38] Gouveia EC Lopes EP Moura I Cruz M Kosminsky L Pernambuco JR Identification of the cut-off value for serum alanine aminotransferase in hepatitis C screening of patients with chronic renal failure on hemodialysis Rev Soc Braz Med Trop 2004 37 1 18 21 10.1590/s0037-86822004000100005 15042176

[B39] Fabrizi F Poordad F Martin P Hepatitis C infection and the patients with end-stage renal disease Hepat 2002 36 1 3 10 10.1053/jhep.2002.34613 12085342

